# Cradle to grave: how social vulnerability correlates with leading causes of injury-related mortality among children and youth

**DOI:** 10.1186/s40621-025-00619-4

**Published:** 2025-09-23

**Authors:** Sarah Gard Lazarus, Sofia Chaudhary, Timothy P. Moran, Terri Miller, Kiesha Fraser Doh, Carlos A. Delgado, Kate Daniels, Chris A. Rees

**Affiliations:** 1 Pediatric Emergency Medicine Associates, PO Box 422002, Atlanta, GA 30342 USA; 2 Children’s Healthcare of Atlanta, 2220 N. Druid Hills Rd. NE, Atlanta, GA 30329 USA; 3 Wellstar Heath System, 793 Sawyer Rd., Marietta, GA 30062 USA; 4 Division of Pediatric Emergency Medicine, Emory University School of Medicine, 100 Woodruff Circle, Atlanta, GA 30322 USA; 5 Emory University School of Medicine, 2015 Uppergate Drive, Atlanta, GA 30322 USA; 6 Georgia Southern University, 1332 Southern Dr, Statesboro, GA 30458 USA; 7 Children’s Healthcare of Atlanta at Scottish Rite, 1001 Johnson Ferry Rd NE., Atlanta, GA 30342 USA

**Keywords:** Injury prevention, Social vulnerability, SUID, Firearm, Drownings

## Abstract

**Background:**

A higher degree of social vulnerability is associated with greater overall injury risk. However, the overlap of social vulnerability with various injury modalities for mortality has been less explored.

**Methods:**

We conducted a cross-sectional study utilizing Georgia death certificates from 2011 to 2021 in youth aged 0–24 years. Mortality rates from firearms, motor vehicle collisions (MVCs), sudden unexpected infant death (SUID), poisonings, and drownings, with census-level social vulnerability index (SVI) categories were evaluated. A negative binomial regression model was created to identify relationships between injury-related cause of death and SVI.

**Results:**

There were 26,362 total deaths from 2011 to 2021 among children and youth. Of these, 10,643 (40%) were due to the top five injury mechanisms causing fatalities in ages 0–24 years in Georgia over the study period. Children and youth with the least advantage in the socioeconomic and minority and language SVIs had higher rates of injury-related mortality from firearm-related deaths, MVCs, and SUID. However, poisonings were most common in the most advantaged quartiles. Differences in number of firearm-related deaths per population were largest in the minority and language status SVI theme.

**Conclusions:**

Children and youth with greater social vulnerability had higher rates of injury-related mortality, except for those due to poisonings. Tailored resources for injury prevention should be focused on least advantaged communities, while poisoning prevention may be best targeted to children and youth in communities with higher SVI. In addition, the impact of systemic investments in healthcare, education, and neighborhood safety on injury-related mortality across SVIs warrants additional investigation.

## Background

Unintentional injuries are the leading cause of child and youth mortality in the United States [1, 2]. Despite outreach, education, and interventions to decrease injuries over the past decade, pediatric injury-related mortality rates have recently increased [3]. Injury-related morbidity and mortality do not affect children equally and are influenced by racial, geographic, and socioeconomic disparities [2, 4–6]. To advocate for legislative, social, and environmental changes, a clear understanding of these disparities is necessary to reduce injury-related mortality among children and youth [4].

The five most common injury-related causes of death in the state of Georgia in the United States among children and youth aged 0–24 years were firearm-related injury, motor vehicle collisions (MVCs), sudden-unexpected infant death (SUID), poisonings, and drownings [1, 7]. These five injury patterns consistently ranked in the top five causes of injury death for individuals aged 0–24 years, based on data from Georgia’s Online Analytical Statistical Information System (OASIS), which compiles information from state death certificates. To date, there has not been a comprehensive exploration of the intersection of geographic, social, and economic disparities in injury-related mortality among children and youth in Georgia. Such an understanding may inform targeted interventions and similar analyses and interventions in other states.

To this end, our objectives were to (1) delineate the common causes of pediatric and young adult injury mortality in Georgia and (2) determine the association between social vulnerability and injury-related mortality among children and youth in Georgia.

## Methods

### Study design

We performed a retrospective analysis of death certificates for children and youth who died from the five leading causes of injury-related mortality in Georgia, including injuries from firearms, motor vehicle collisions (MVCs), sudden unexpected infant death (SUID), poisoning, and drownings [1, 7]. For SUID, we included children aged < 12 months as this injury is specific to infants [8]. For other injury modalities, we included children and youth aged 0–24 years who died in Georgia from January 1 st _,_ 2011 through December 31 st, 2021. All relevant variables, including race and location of death were consistently recorded. No records contained missing or incomplete data. We focused our analyses on the five most common causes of injury-related death to assess the relationship between social vulnerability and the most common causes of death and inform potential interventions to prevent future injury-related deaths. We followed the Strengthening the Reporting of Observational Studies in Epidemiology (STROBE) reporting guidelines. The study was deemed exempt from ethical review by Children’s Healthcare of Atlanta due to the use of de-identified and publicly available data.

### Study setting

The state of Georgia is located in the southeastern United States. Georgia’s population is approximately 10.7 million [8]. Georgia has 159 counties with a mixture of urban and rural designations (120 [75.4%] are rural, yet only 21% of the state’s population lives in a rural area) [9]. Overall, 13.6% of inhabitants live in poverty. Of Georgia’s population, 59% identify as White, 33% as Black, 5% as Asian, 3% as Mixed Race, < 1% as Native Hawaiian/Pacific Islander, and < 1% as American Indian [10]. The KIDS COUNT^®^ Data Book uses 16 indicators to rank each state across four domains: health, education, economic well-being, and family/community to assess well-being [11]. Georgia ranked 37th overall for child well-being, 32nd in economic well-being, 31 st in education, 43rd in health, and 42nd in family/community (i.e., a composite measure of children living in high-poverty areas, teen births, and children in single-parent families) [12].

### Variables

Data from Georgia death certificates were obtained from the Georgia Department of Public Health. Data for denominators to determine the number of injury-related deaths were obtained from the US census tract utilizing the 2020 American Community Survey [9]. Georgia death certificates were reviewed for basic demographic data such as sex, race, and the decedent’s age and place of residence at the time of death, which was then used to evaluate the decedent’s Social Vulnerability Index (SVI). SVI utilizes census-tract level data to determine markers of social vulnerability and has historically been used for disaster preparedness for 16 factors, such as poverty, lack of vehicle access, and crowded housing [13]. These 16 factors are then grouped into four themes: (1) socioeconomic status (i.e., rates of poverty/unemployment, income, and educational attainment), (2) household composition/disability (i.e., single-parent households, percentage of persons ≥ 65 years or < 18 years of age), (3) minority status/language (i.e., percentages of individuals who are members of racial/ethnic minority groups), and (4) housing type/transportation (i.e., housing in structures with > 10 units, mobile homes, households without vehicles) [14]. SVI has been shown to be impactful in the evaluation of various injury patterns throughout the United States [5, 13, 15–19].

### Analyses

Decedent demographics were compared using chi-square (or Fisher’s exact tests when appropriate) and two sample t-tests to assess for homogeneity of the included population. Non-parametric tests (i.e., Wilcoxon rank-sum tests) were used for data that were not normally distributed. Descriptive statistics were summarized using means, standard deviations or medians, and interquartile ranges for continuous data. Counts and percentages were used for categorical data. Using these methods, bias was minimized. For SVI, we evaluated the four central themes and 16 associated variables to determine associations between the mortality mechanism and the four SVI themes [14]. A negative binomial regression model was created to identify relationships between the outcome of injury-related number of deaths per population and SVI quartiles by each of the four SVI themes. Comparisons in the negative binomial regression model were adjusted for all included components of SVI scales. Statistical analysis was completed using SAS 9.4 (Cary, NC).

## Results

There were 26,362 deaths in children and youth aged 0–24 years from 2011 to 2021 with 10,643 (40%) being due to the top 5 injury-related causes and were thus included in our analyses. All deaths eligible were included for analysis. Most injury-related deaths were in youth aged 15–24 years (*n* = 7,662, 72%; Table 1). The median age of those affected by firearms was 20 years (interquartile range [IQR] 18, 22), for MVCs 20 years (IQR 16, 22), for poisonings 22 years (IQR 20, 23), and for drownings 9 years (IQR 3, 18). All injury-related mechanisms of death affected males (*n* = 7,845, 74% of overall deaths) more than females, with the greatest difference in firearm-related mortality (male 87%, female 13%). Injury-related mortality affected Non-Hispanic Black children (49%) and White children (49%) almost equally, followed by Asian children (1%) and Multi-Racial children (1%) (Table 1). Both Native-American and Pacific-Islander children made up only approximately 0.01% of all deaths. SUID and firearm injury-related deaths affected Black children and youth more than other races (i.e., 56% of SUID deaths and 66% of firearm-related deaths occurred among Black children and youth). White children comprised the majority of drowning-related deaths (53%), MVC-related deaths (59%), and poisoning-related deaths (82%).

**Table 1:** Demographics and injury characteristics for included injury-related deaths (*N*=10,643)

	Total	Drowning	Poison/Noxious Exposure	Firearm	MVC	SIDS
**Age groups**						
<1 year	1811 (17)	12 (2.3)	5 (0.4)	5 (0.1)	52 (1.5)	1737 (100)
1–4 years	425 (4)	184 (34.7)	14 (1.2)	42 (1.1)	185 (5.4)	0 (0)
5–9 years	305 (2.9)	75 (14.1)	9 (0.8)	42 (1.1)	179 (5.2)	0 (0)
10–14 years	440 (4.1)	52 (9.8)	13 (1.1)	164 (4.3)	211 (6.2)	0 (0)
15–24 years	7662 (72)	208 (39.2)	1122 (96.5)	3545 (93.3)	2787 (81.6)	0 (0)
**Sex**						
Female	2798 (26.3)	121 (22.8)	330 (28.4)	493 (13)	1064 (31.2)	790 (45.5)
Male	7845 (73.7)	410 (77.2)	833 (71.6)	3305 (87)	2350 (68.8)	947 (54.5)
**Race**						
Native American	5 (<0.01)	0 (0)	1 (0.1)	2 (0.1)	2 (0.1)	0 (0)
Asian	147 (1.4)	13 (2.4)	15 (1.3)	49 (1.3)	54 (1.6)	16 (0.9)
Black	5166 (48.5)	226 (42.6)	183 (15.7)	2499 (65.8)	1289 (37.8)	969 (55.8)
Multiracial	114 (1.1)	9 (1.7)	7 (0.6)	30 (0.8)	33 (1)	35 (2)
Pacific Islander	14 (0.1)	1 (0.2)	0 (0)	4 (0.1)	7 (0.2)	2 (0.1)
White	5197 (48.8)	282 (53.1)	957 (82.3)	1214 (32)	2029 (59.4)	715 (41.2)
**Ethnicity**						
Hispanic	817 (7.7)	64 (12.1)	77 (6.6)	213 (5.6)	365 (10.7)	98 (5.6)
Non-Hispanic	9815 (92.2)	467 (87.9)	1086 (93.4)	3582 (94.3)	3042 (89.1)	1638 (94.3)
Unknown	11 (0.1)	0 (0)	0 (0)	3 (0.1)	7 (0.2)	1 (0.1)
**Cause of Death**						
Drowning	531 (5)	–	–	–	–	–
Firearm	3798 (35.7)	–	–	–	–	–
Motor vehicle collision	3414 (32.1)	–	–	–	–	–
Poisoning	1163 (10.9)	–	–	–	–	–
Sudden unexplained infant death	1737 (16.3)	–	–	–	–	–

### Differences in Injury-Related deaths by SVI

In each of the four SVI themes, there were higher mortality rates among the most socially vulnerable groups (*P* < 0.001, Table 2). The SVI socioeconomic status theme had the largest disparities between those least and most advantaged, with 18.1% of deaths (*n* = 1,925) among the most advantaged quartile and 32% of deaths (*n* = 3,441) among the most disadvantaged quartile. This was closely followed by the minority status/language SVI, with 20.7% of deaths (*n* = 2,202) among the most advantaged quartile, and 34.8% of deaths (*n* = 3,703) from the most disadvantaged quartile. In the household composition/disability SVI, 18.9% of deaths (*n* = 2,015) occurred among the most advantaged quartile and 31.3% of deaths (*n* = 3,329) occurred among the most disadvantaged quartile (Table 2).

**Table 2:** Comparison of the proportion of injury-related deaths by social vulnerability index (SVI) themes

SVI Theme	*n* (%)	*P* value
*Socioeconomic (N=10,643)*		<0.001
Lowest quartile (most advantaged)	1,925 (18.1)	
Second quartile	2,415 (22.7)	
Third quartile	2,862 (26.9)	
Fourth quartile (most disadvantaged)	3,441 (32.3)	
*Household Composition/Disability (N=10,643)*		<0.001
Lowest quartile (most advantaged)	2,015 (18.9)	
Second quartile	2,577 (24.2)	
Third quartile	2,722 (25.6)	
Fourth quartile (most disadvantaged)	3,329 (31.3)	
*Minority/Language (N=10,643)*		<0.001
Lowest quartile (most advantaged)	2,202 (20.7)	
Second quartile	2,301 (21.6)	
Third quartile	2,437 (22.9)	
Fourth quartile (most disadvantaged)	3,703 (34.8)	
*Housing Type/Transportation (N=10,643)*		<0.001
Lowest quartile (most advantaged)	2,127 (20.0)	
Second quartile	2,576 (24.2)	
Third quartile	2,867 (26.9)	
Fourth quartile (most disadvantaged)	3,073 (28.9)	

### Firearm-Related deaths

**Fig. 1:**
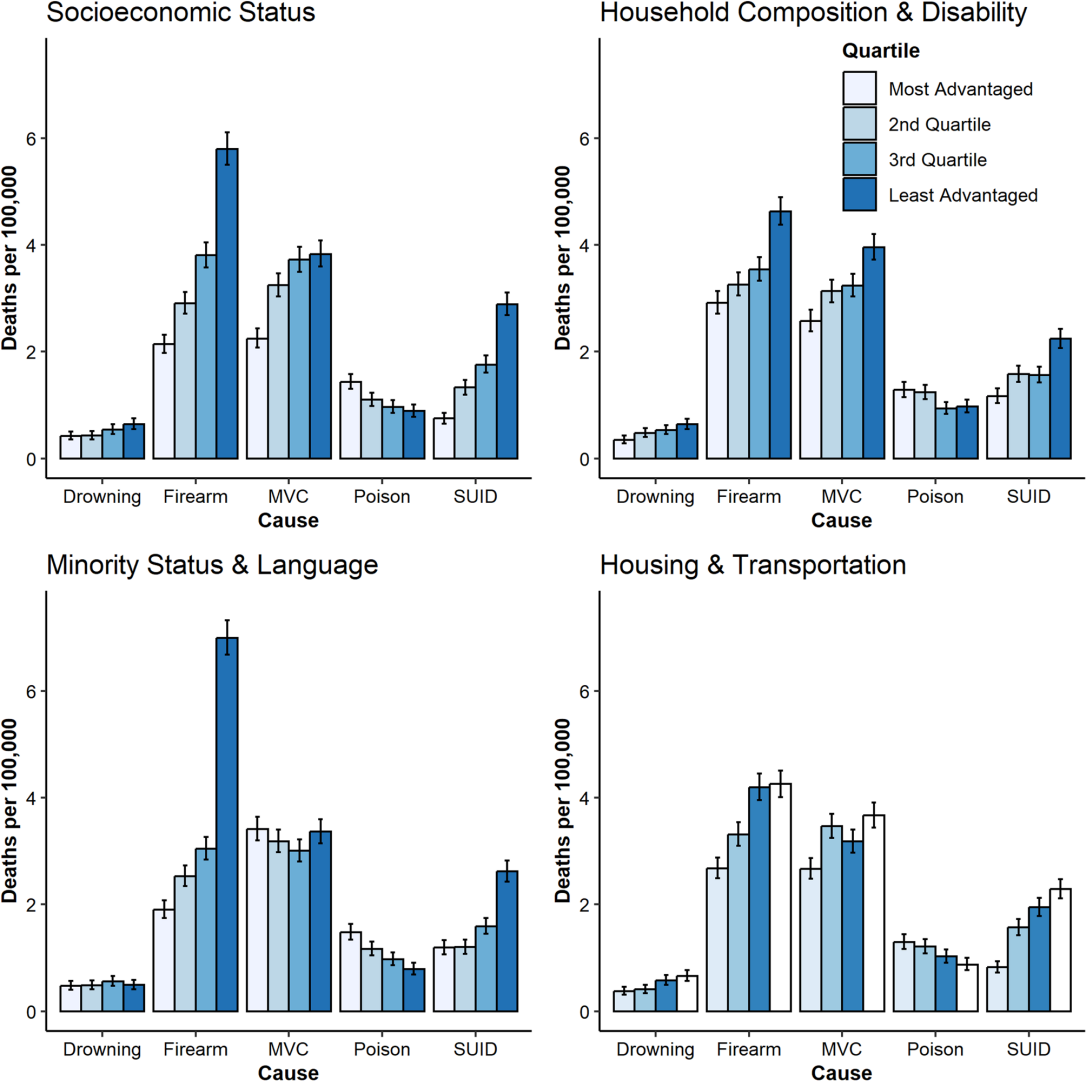
Unadjusted incident injury-related deaths by injury mechanism and social vulnerability index quartile

Firearm-related deaths were the most common type of injury mortality (*n* = 3,798, 36% of all injury-related deaths). The unadjusted number of firearm-related deaths per population was larger in the least advantaged quartile population in each of the four SVI themes (Fig. 1). The minority status and language theme had the largest difference in the number of firearm-related deaths per population between the least and most advantaged quartiles. However, in adjusted analyses, firearm-related deaths was only more common in the least advantaged groups by socioeconomic and minority status/language SVI themes (Table 3).

**Table 3:** Negative binomial regression models to assess the association between incident injury-related deaths and social vulnerability index themes and quartile*

		Socioeconomic		Household composition and disability		Minority Status and Language		Housing and Transportation	
		Rate Ratio (95% CI)	*P* value	Rate Ratio (95% CI)	*P* value	Rate Ratio (95% CI)	*P* value	Rate Ratio (95% CI)	*P* value
Firearms	Most advantaged	*Referent*		*Referent*		*Referent*		*Referent*	
	Quartile 2	1.55 (1.27–1.89)	**<0.001**	1.04 (0.89–1.22)	0.63	3.06 (2.60–3.61)	**<0.001**	1.00 (0.84–1.19)	0.99
	Quartile 3	1.35 (1.14–1.61)	**<0.001**	0.94 (0.80–1.09)	0.4	1.45 (1.23–1.70)	**<0.001**	1.1 (0.93–1.30)	0.25
	Least advantaged	1.16 (0.99–1.37)	0.07	0.98 (0.84–1.14)	0.76	1.30 (1.11–1.53)	**0.001**	1.07 (0.91–1.25)	0.43
Motor Vehicle Collisions	Most advantaged	*Referent*		*Referent*		*Referent*		*Referent*	
	Quartile 2	1.76 (1.45–2.15)	**<0.001**	1.34 (1.14–1.58)	**<0.001**	0.77 (0.65–0.90)	**0.001**	1.00 (0.84–1.19)	0.98
	Quartile 3	1.70 (1.43–2.02)	**<0.001**	1.17 (1.00–1.36)	0.05	0.74 (0.64–0.87)	**<0.001**	0.93 (0.79–1.09)	0.37
	Least advantaged	1.46 (1.25–1.70)	**<0.001**	1.18 (1.02–1.38)	**0.03**	0.89 (0.77–1.03)	0.11	1.10 (0.94–1.29)	0.22
Sudden Unexplained Infant Deaths	Most advantaged	*Referent*		*Referent*		*Referent*		*Referent*	
	Quartile 2	2.60 (2.00–3.30)	**<0.001**	1.09 (0.90–1.33)	0.38	1.40 (1.15–1.71)	**<0.001**	1.59 (1.27–1.99)	**<0.001**
	Quartile 3	1.79 (1.43–2.25)	**<0.001**	0.97 (0.80–1.18)	0.74	1.02(0.84–1.24)	0.86	1.57 (1.27–2.0)	**<0.001**
	Least advantaged	1.52 (1.23–1.88)	**<0.001**	1.16 (0.96–1.41)	0.13	0.90 (0.74–1.10)	0.31	1.53 (1.24–1.89)	**<0.001**
Poisonings	Most advantaged	*Referent*		*Referent*		*Referent*		*Referent*	
	Quartile 2	0.89 (0.68–1.16)	0.39	0.95 (0.77–1.19)	0.67	0.59 (0.47–0.74)	**<0.001**	0.86 (0.67–1.09)	0.2
	Quartile 3	0.81 (0.65–1.01)	0.06	0.85 (0.69–1.04)	0.12	0.71 (0.58–0.86)	**<0.001**	0.95 (0.76–1.18)	0.63
	Least advantaged	0.83 (0.68–1.01)	0.06	1.04 (0.86–1.27)	0.67	0.80 (0.67–0.97)	**0.02**	1.03 (0.84–1.26)	0.78
Drownings	Most advantaged	*Referent*		*Referent*		*Referent*		*Referent*	
	Quartile 2	1.11 (0.77–1.59)	0.59	1.58 (1.12–2.15)	**0.004**	0.77 (0.57–1.04)	0.09	1.56 (1.12–2.16)	**0.008**
	Quartile 3	1.00 (0.73–1.37)	0.99	1.40 (1.04–1.89)	**0.03**	0.94 (0.71–1.24)	0.67	1.44 (1.06–1.95)	**0.02**
	Least advantaged	0.89 (0.66–1.20)	0.45	1.35 (1.00–1.82)	0.05	0.90 (0.68–1.18)	0.44	1.06 (0.78–1.44)	0.73

Clinically significant with a *p*-value of ≤0.05 are shown in bold

*Models adjusted for all included components of social vulnerability index scales

### MVC-related deaths

MVC-related deaths were the second most common cause of injury-related deaths (*n* = 3,414, 32%). The number of MVC-related deaths per population was higher in the least advantaged quartiles for all SVI themes except for the minority status/language theme in unadjusted comparisons (Fig. 1). In adjusted analyses, the number of MVC-related deaths per population was higher in less advantaged quartiles in the socioeconomic theme and the household composition/disability SVI theme (Table 3). However, the adjusted number of deaths per population from MVCs was less common in more advantaged quartiles in the minority status and language theme. The number of MVC-related deaths per population did not differ by quartile in analyses of the housing and transportation SVI theme.

### SUID

There were 1,737 deaths from SUID, comprising 16% of the total injury-related deaths. The number of SUIDs per population was higher in the least advantaged quartiles across all four SVI themes in unadjusted comparisons (Fig. 1). The number of SUIDs per population was higher in less advantaged quartiles by the socioeconomic and housing and transportation SVIs, but the same pattern was not observed in adjusted analyses in the household composition/disability SVI and the minority status and language SVI (Table 3).

### Poisoning-related deaths

There were 1,163 poisoning deaths, comprising 11% of all injury-related deaths. Poisoning-related deaths defied the trends of other injury-related deaths as the unadjusted number of deaths per population from poisonings was higher in the most advantaged quartiles in each of the four SVI themes (Fig. 1). However, this relationship was lost in adjusted analyses except for the minority status and language SVI theme, in which adjusted poisoning-related deaths were less common among more disadvantaged quartiles (Table 3).

### Drowning-related deaths

Drowning-related deaths comprised only 5% (*n* = 531) of injury-related deaths included in our analyses. The unadjusted number of drowning deaths per population was higher among children and youth in the least advantaged quartiles in all SVI themes except for the minority status and language SVI, in which the unadjusted drowning-related deaths did not differ substantially among the quartiles (Fig. 1). Like most injury-related deaths, drowning-related deaths were more common in less advantaged quartiles in the household composition and disability and housing and transportation SVI themes (Table 3).

## Discussion

Overall, in our analyses that included > 10,000 deaths among children and youth, when looking at the unadjusted number of injury-related deaths per population, there were more deaths among those from the most disadvantaged quartiles in all SVI themes in the state of Georgia. This was especially the case among the socioeconomic and minority status/language themes. These differences, however, did not necessarily follow a dose-response by quartile. Poisonings, in contrast, were more common among more advantaged groups.

Our study is the first, to our knowledge, to evaluate mortality among children and youth from five of the most common injury mechanisms by SVI. Drowning and poisonings are infrequently referenced in injury prevention literature. Other studies have focused on multiple injury mechanisms in ages 1–45, but did not separate injury-related mortality by mechanism to determine the SVIs most associated specific injury mechanisms and did not focus specifically on children and youth injuries [16]. A more granular assessment of injury-related number of deaths per population can inform targeted injury prevention approaches for children and youth.

Since 2017, firearm-related injuries have shown a substantial increase, becoming the leading cause of death for children and youth aged 0–24 years [20]. Our findings from the state of Georgia aligned with this disturbing trend. Moreover, our findings are similar to other emerging literature showing a strong relationship between greater social vulnerability and greater risk of firearm-related injuries and deaths [5, 6, 15, 16, 18]. Our study expands these findings by providing a more granular view of which aspects of social vulnerability may place children and youth at risk for greater risk of firearm-related deaths. We found that children and youth in the least advantaged minority status and language SVI had the greatest rates of number of deaths per population due to firearms. Other studies have demonstrated that Black male youth are as much as 18 times more likely to die from firearm-related violence than White youth [21]. The reasons for this disparity are likely multifactorial, but residential segregation could lead to increased firearm violence, even in segregated areas without income disparities [22]. Number of deaths per population due to firearms also differed by the socioeconomic SVI quartiles. This aligns with other studies that suggest counties with the highest levels of poverty also have highest rates of youth firearm homicides, suicides, and unintentional firearm deaths [6]. Inequities begin early in life and early childhood development programs, more generous health policies, enhancing employment opportunities, improved wage, and civil rights policies and equity-focused legislation may help address racial and socioeconomic disparities, which in turn may reduce firearm-related deaths among children and youth [23].

MVC deaths have decreased approximately 40% from 2000 to 2020 [20]. However, these deaths are not equally distributed across all groups. In our study, MVC-related deaths were most common among children and youth in the least advantaged socioeconomic SVIs. Prior research suggests that children with less socioeconomic advantage may be less likely to be properly restrained [24]. However, we also observed higher rates of MVC-related deaths among more advantaged children and youth in the housing/transportation theme and minority status/language theme (Fig. 1). Racial and socioeconomic factors play a part in whether a teen is given opportunities to drive. Previous studies suggest that teens are less likely to drive when they are from households with less financial resources for a vehicle, driver’s education, and automobile insurance. Thus, more socioeconomically advantaged children may drive at a younger age [25]. In addition, as ride-sharing increases, there are inconsistent recommendations and laws regarding restraint use in ride-sharing programs, with an unclear impact on MVC deaths [26, 27]. Further studies are warranted to understand the reasons for inconsistent rates of MVC-related deaths among both advantaged and disadvantaged groups.

SUID is a leading cause of death in children aged < 1 year and is strongly associated with modifiable sleep environments. Since 1999, rates of SUID have remained relatively unchanged, and vary widely by state [8, 28]. Similar to prior studies that suggest that child abuse-related fatalities are more common among socioeconomically disadvantaged populations, our study showed that children in the least advantaged socioeconomic group had the highest rates of SUID-related deaths compared to those in the most advantaged socioeconomic SVI quartile [29]. Findings from Georgia suggest that Medicaid-enrolled families have four times greater risk of SUID than children with any other insurance type [7]. Furthermore, prior studies suggest that SUID rates are twice as high among Black infants compared to White infants, and in some states has been reported to be as high as 12x that of White infants [30, 31]. In at least one other study, factors such as education level and insurance status, along with race, were found to significantly influence SUID rates, with individuals who had lower educational attainment or public insurance experiencing higher rates of SUID. Urbanicity, prematurity status and maternal complications had less of an impact on SUID rates [32]. Disparities have widened following the COVID-19 pandemic, with even larger increases in SUID for infants of mothers who are publicly insured, have attained less education, and non-Hispanic Black mothers [33]. Although our study was not designed to elucidate reasons for such disparities, concerted efforts to promote safe sleeping patterns are urgently needed for high-risk groups to prevent future SUID.

Regarding poisonings, other studies suggest that population density (housing type and transportation), minority status, and household composition/disability are most associated with poisoning-related deaths, with those least advantaged most likely to die from fatal drug overdoses than those who lived in more advantaged neighborhoods [34]. Our study differs this previous finding. To our knowledge, there is only one previous study demonstrating that poisoning-related deaths may be more common in certain affluent populations [35]. Greater mortality in those of higher advantage could be due to increased prescription medication access due to more liberal prescription practices in children of higher advantage, which eventually can increase the risk of use and misuse, or that alcohol and marijuana use may be associated with higher economic status, which may be a gateway for opioids or other more lethal drugs [36]. Ultimately, additional studies are warranted to elucidate the reason that poisoning-related mortality was most common among more advantaged populations in Georgia. It is important to track future overdose deaths and to determine if these poisonings were intentional or not to inform further interventions.

### Limitations

Our study had several limitations. We reviewed the SVI where the patient’s address was on the death certificate, not the location of death, which may not reflect residence at the time of death. Although the SVI provides a multifaceted assessment of social well-being, it does not provide granularity on what elements in the household or environment may be driving such disparities in injury-related deaths. In addition, because our data utilized death certificate data which may not always involve a complete diagnostic autopsy, the true cause of death may not have always been captured. However, as the included causes of death are injury-related, discrepancies between death certificate and the actual causes of death may have been less frequent. Our study is limited to state-wide data and not a national dataset, and only assessed SVI and basic demographics, and not the overall community breakdown, and could not account for other unmeasured behavioral, social, and environmental factors that may contribute to injury-related mortality. However, these methods can be adapted for a multi-state or national analysis. Lastly, it is important to note that although race and ethnicity comprise an SVI theme, racial disparities are not the result of race itself, but are shaped by institutional and historical injustices and our findings should be interpreted with the acknowledgement of race as a social construct [37].

## Conclusions

Our results support previous studies showing that SUID and firearm-related mortality are more common among children and youth who live in more disadvantaged neighborhoods. Conversely, poisonings were more common among children and youth who lived in more advantaged neighborhoods. Further exploration of shared risk and protective factors across social vulnerability themes and injury-related mortality could help identify key opportunities for policy changes and targeted interventions to reduce mortality and morbidity in children and youth from preventable causes of death.

## Data Availability

All data used in this study can be accessed through reasonable request to the Georgia Department of Public Health, Office of Health Indicators for Planning; Centers for Disease Control and Prevention, Agency for Toxic Substances and Disease Registry and US census bureau.

## Abbreviations

MVC: Motor vehicle collision
SUID: Sudden unexpected infant death
SVI: Social vulnerability index

## Declarations

### About this supplement

This article has been published as part of Injury Epidemiology Volume 12 Supplement 1, 2025: Proceedings of the 29th Annual Injury Free Coalition for Kids^®^ Conference: Forging New Frontiers: Engineering for Equity. The full contents of the supplement are available online at https://injepijournal.biomedcentral.com/articles/supplements/volume-12-supplement-1.
